# Exome sequencing identifies novel somatic variants in African American esophageal squamous cell carcinoma

**DOI:** 10.1038/s41598-021-94064-0

**Published:** 2021-07-20

**Authors:** Hayriye Verda Erkizan, Shrey Sukhadia, Thanemozhi G. Natarajan, Gustavo Marino, Vicente Notario, Jack H. Lichy, Robert G. Wadleigh

**Affiliations:** 1 Institute for Clinical Research, Veterans Affairs Medical Center, Washington, DC USA; 2 Dartmouth-Hitchcock Medical Center, Lebanon, NH USA; 3 Queromatics, Laveen, AZ USA; 4 Hepatology and Gastroenterology, Veterans Affairs Medical Center, Washington, DC USA; 5 Lombardi Comprehensive Cancer Center, Georgetown University, Washington, DC USA; 6 Pathology and Laboratory Service, Veterans Affairs Medical Center, Washington, DC USA; 7 Hematology and Medical Oncology, Veterans Affairs Medical Center, Washington, DC USA

**Keywords:** Cancer, Cancer genomics, Oesophagus, Cancer genomics, Oesophageal cancer

## Abstract

Esophageal cancer has a strikingly low survival rate mainly due to the lack of diagnostic markers for early detection and effective therapies. In the U.S., 75% of individuals diagnosed with esophageal squamous cell carcinoma (ESCC) are of African descent. African American ESCC (AA ESCC) is particularly aggressive, and its biological underpinnings remain poorly understood. We sought to identify the genomic abnormalities by conducting whole exome sequencing of 10 pairs of matched AA esophageal squamous cell tumor and control tissues. Genomic analysis revealed diverse somatic mutations, copy number alterations (SCNAs), and potential cancer driver genes. Exome variants created two subgroups carrying either a high or low tumor mutation burden. Somatic mutational analysis based on the Catalog of Somatic Mutations in Cancer (COSMIC) detected SBS16 as the prominent signature in the high mutation rate group suggesting increased DNA damage. SBS26 was also detected, suggesting possible defects in mismatch repair and microsatellite instability. We found SCNAs in multiple chromosome segments, encoding *MYC* on 8q24.21,* PIK3CA* and *SOX2* on 3q26, *CCND1*, *SHANK2*, *CTTN* on 11q13.3, and *KRAS* on 12p12. Amplifications of *EGFRvIII* and *EGFRvIVa* mutants were observed in two patients, representing a novel finding in ESCC that has potential clinical relevance. This present exome sequencing, which to our knowledge, represents the first comprehensive exome analysis exclusively in AA ESCC, and highlights novel mutated loci that might explain the aggressive nature of AA ESCC and lead to the development of diagnostic and prognostic markers as well as therapeutic targets.

## Introduction

Esophageal cancer (EC) is one of the most lethal cancers, primarily due to the lack of early diagnostic markers and effective treatments. EC represents 26.3% of aerodigestive malignancies in the U.S., thus imposing a substantial burden on a sizeable proportion of the population and the health care system^1^. In 2021, the ratio of expected deaths (15,530 cases) to expected new diagnoses (19,260 cases) is estimated to be 80%, and men are 3.5 times more likely to be diagnosed with EC than women^1^. EC is the seventh leading cause of cancer deaths in males, accounting for 4% of all cancer deaths^1^. For all stages, combined survival of esophageal cancer is comparable to liver cancer (20%) and slightly higher than pancreas cancer (10%), which has one the lowest survival rates among diverse cancers^1^.

EC consists of two major histological subtypes, esophageal adenocarcinoma (EAC) and ESCC; each subtype predominantly affects a specific population. AA comprise about 75% of ESCC patients^2,3^. The 5-year survival rate for localized EC in AA patients has been found to be 25%, in contrast the survival rate for EC patients of Caucasian origin is 48%^4^. However, for metastatic disease, the survival rate decreases dramatically to 5% regardless of race^2^. The lack of access to quality health care and low socio-economic status have been reported to partly contribute to the higher incidence and mortality rates of ESCC among AA^5,6^.

EC is caused by the combined effects of genetic and environmental risk factors, including tobacco use, alcohol consumption, and certain dietary habits^7^. Various EC studies have disclosed gender, racial, socioeconomic, regional disparities, and variability in worldwide geographical incidence rates^7,8^. ESCC is endemic in parts of Asia such as China, India, Iran, South Africa, and South American countries^9^.

Various studies revealed molecular evidence for the heterogeneous nature of ESCC^10^, yielding three main molecular subgroups^11^. Group 1 (ESCC1), or the “classical” subtype, closely resembles head and neck and lung squamous cell carcinomas^11^, and has been shown to be the most common in Asian patients. ESCC1 is typically associated with mutations in the genes involved in oxidative stress and detoxification pathways, and the amplification of *SOX2* and *TP63*. Group 2 (ESCC2), occurs primarily in Eastern European and South American patients that carry frequent mutations in *NOTCH1*, *ZNF750*, *KDM6A*, and *KMT2D* ^11^. Group 3 (ESCC3) has been found in very few patients, for example, one study reported five AA patients that displayed, activation of PI3K pathway, and *SMARCA4* mutations and relatively low occurrence of *TP53* mutations^11^. These subtypes highlight dis-similarities that exist between Asian, Caucasian, and AA ESCC^12–14^.

Defects in molecular and genetic mechanisms associated with AA ESCC are not well defined due, in part, to its under-representation in epidemiological studies^11,12,15^. Hence, the biological basis for the lethality and aggressiveness of AA ESCC remains to be fully understood^16^. In our earlier studies, we conducted the first comparative genomic hybridization (CGH) analysis in AA ESCC that revealed widespread chromosomal imbalances and prominent abnormalities throughout the AA ESCC genome^17^. We performed gene expression profiling in AA ESCC tumors, which revealed a profound disruption of genes involved in various pathways including stress response and detox pathway, integrin signaling, and protein ubiquitination^18^. This study identified uniquely impaired biological processes in AA ESCC, which partially overlapped with findings in ESCC patients of Asian origin^18^.

In our current study, we sought to identify somatic mutations in the AA ESCC genome by whole-exome sequencing (WES). We identified two subgroups of ESCC based on tumor mutation burden (TMB) and revealed recurrent novel SCNAs in cancer-related genes. We performed analysis based on COSMIC and detected signatures SBS16 and SBS26 that might contribute to AA ESCC pathogenesis. These deleterious alterations may play a role in the aggressive nature of the disease in the AA population. Further analysis in a larger set of AA ESCC may lead to the identification of AA ESCC specific clinical biomarkers and therapeutic targets.

## Result

### Whole-exome sequencing reveals a complex mutation profile, and SCNAs in AA ESCC

Whole-exome sequencing was performed on matched normal-tumor samples from 10 AA patients (nine males and one female) with advanced-stage ESCC, with ages ranging from 53 to 80 years (Supplementary Table S1). All patients, except for one, reported tobacco use and alcohol consumption. We employed an analysis pipeline delineated in Supplementary Fig. S1 that involved rigorous quality control (QC) and filtering mechanisms, along with methods described in the Genome Analysis Toolkit (GATK) Best Practices (https://gatk.broadinstitute.org/hc/en-us). Somatic single nucleotide variants (SNVs) and short insertion-deletions (InDels) called by at least two of three variant calling algorithms were filtered by a read-depth of 50 × or higher. This analysis included variant allele frequency (VAF) > 5% and rare variants with < 1% minor allele frequency (MAF) in African population (Supplementary Fig. S1).

**Figure 1:**
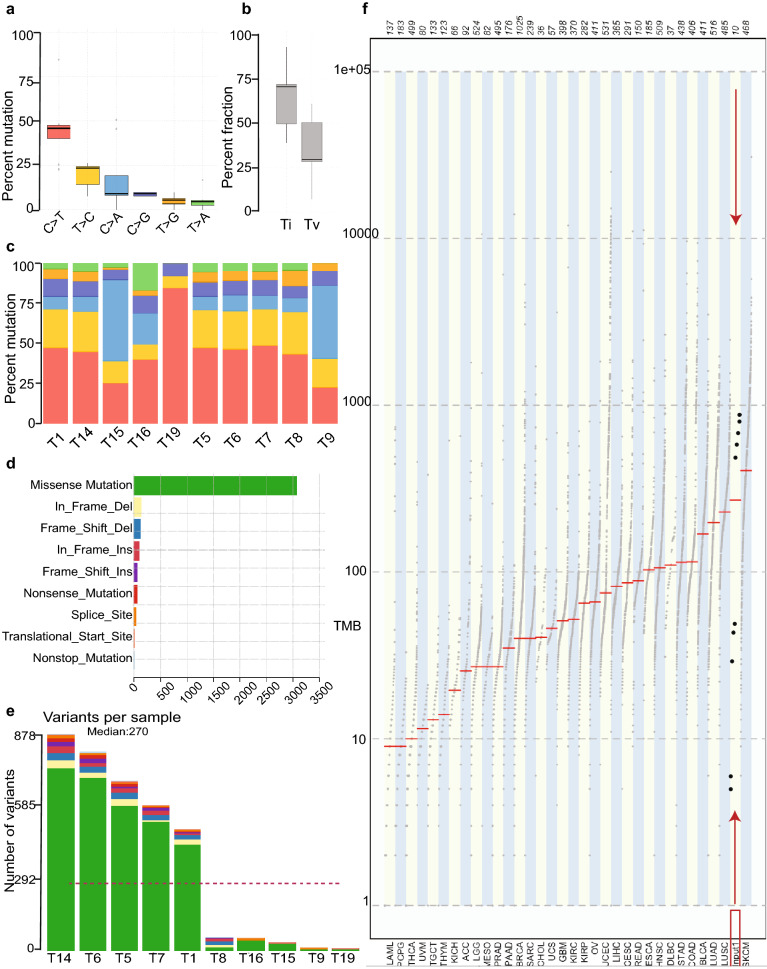
Summary of exome sequencing and tumor mutation burden (TMB). (**a**) Proportion of somatic substitution types in processed and filtered SNVs. (**b**) The ratio of somatic transition to transversion of AA ESCC cohort. (**c**) The proportion of somatic substitution types (C > T, red, T > C, yellow, C > A, blue, C > G, purple, T > G, orange, T > A, green) in each tumor samples. (**d**) The proportion of mutation types in the samples, missense mutations (green), in frame deletions (yellow), frameshift deletions (blue), in frame insertions (brick red), frameshift insertions (purple), nonsense mutations (bright red), changes in the splice sites (orange), and nonstop mutations (light blue). (**e**) Number and types of variants found in each sample. (**f**) The comparison of mutation burden of AA ESCC pilot cohort across TCGA datasets. Our samples are indicated by red rectangle and arrows. Y-axis is the total number of non synonymous mutations found in each tumor sample.

We identified predominantly C > T substitution in the majority of the samples (Fig. 1a). Transition to transversion ratio (Ti/Tv) was 2.4 (Fig. 1b). Samples T9 and T15 demonstrated significant C > A transversion (Fig. 1c). Missense mutations (N = 3682) constituted the primary type of alteration in the coding region of tumor samples (Fig. 1d). Nonstop (stop-loss) was rare (N = 23). A median of 270 variants per sample was revealed in our cohort (Fig. 1e) (Supplementary Table S2). Five samples (T1, T6, T5, T7, and T14) displayed a high tumor mutation burden (TMB) referred to as High Mutation Rate (HMR) samples. In each of these samples, nonsynonymous mutations ranged from 377 to 813 (Table 1). In contrast, low TMB or Low Mutation Rate (LMR) was detected in T8, T9, T15, T16, and T19, each sample showed a median of 29 (ranging from 5 to 44) nonsynonymous coding mutations (Table 1).

**Table 1:** Number of mutations* in each tumor sample.

Tumor	2 or more variant caller (> 50DP, 5 > VAF)		
	#SNV < 1%AFR	# of InDel	Total coding mutations
T14	3460	1940	878
T6	3339	1239	804
T5	2716	1243	686
T1	2152	1033	588
T7	1663	956	491
T16	125	9	49
T15	80	1	44
T8	93	629	29
T9	22	0	6
T19	13	0	5

*Mutations called by two or more algorithms, filtered for rare (< 1% MAF) African population frequency, and sorted by somatic nonsynonymous coding mutations.

Comparison of the mutation rate of the AA ESCC cohort with the TMB of diverse types of cancer in the TCGA database showed that the median mutation rate of AA ESCC was greater than most tumors, which ranked between skin cutaneous melanoma and lung squamous cell carcinoma (Fig. 1f).

**Figure 2:**
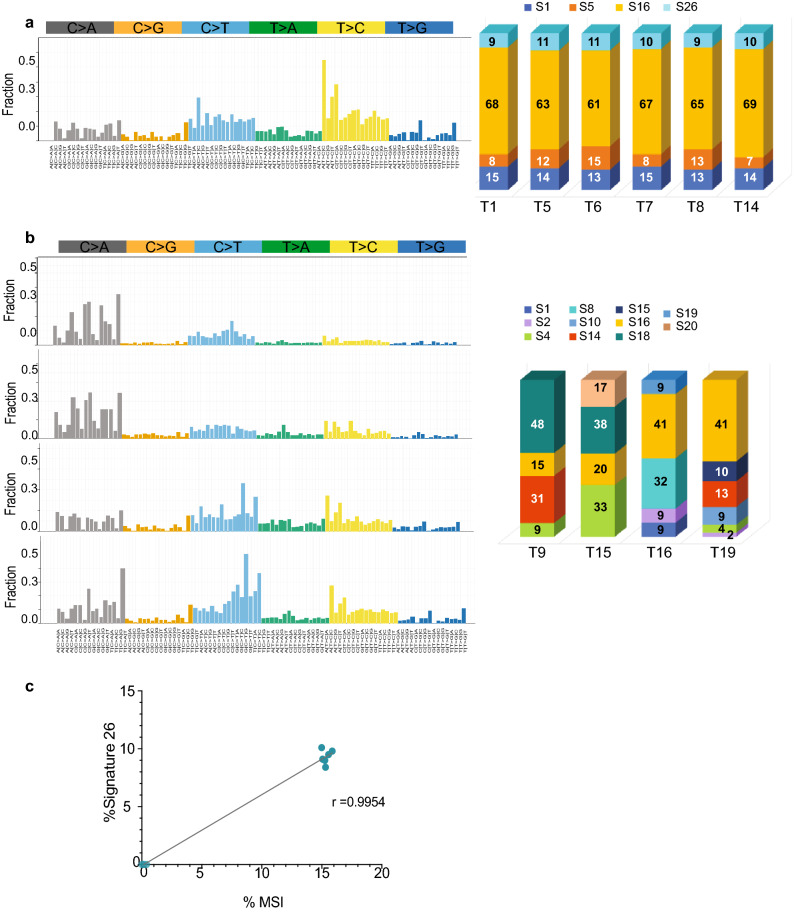
Mutational Signatures based on COSMIC Signatures extrapolate the nature of mutational processes in AA ESCC. (**a**) T > C mutations at ATN trinucleotides constitute Signature 16, which is demonstrated by yellow color in the bar graphs showing the fraction of mutational signatures contributed to whole mutations in each tumor sample. Signatures 1, 5, 16 and 26 were found in similar proportions (percentage) across the samples of T1, T5, T6, T7, T8, and T14. (**b**) Samples T9, T15, T16, and T19 contained various mutational signatures, each tumor sample had a unique mutational signature combination. Bar graphs shows the percentage of each signature in the tumor samples. (**c**) Signature 26 was correlated with percent microsatellite instability (%MSI) in Signature 16-high samples. Pearson correlation coefficient *r* was 0.9954 and 95% confidence interval 0.9799 to 0.9989. R^2^ was calculated as 0.9908.

We performed somatic mutational signature analysis^19^ based on COSMIC and identified SBS16 in all our AA ESCC study samples, thus representing the predominant mutational signature (Fig. 2a, b, yellow portion of the bar graphs). Eight tumor samples displayed the highest contribution to SBS16 (Fig. 2a, b). SBS16 may be generated by inefficient nucleotide excision repair and elevated levels of DNA damage suggesting the involvement of these mechanisms in AA ESCC^19^. In addition to S16, Signatures 1, 5, and 26 were observed in the HMR samples (Fig. 2a). SBS1 suggests failure to repair the product of spontaneous or enzymatic deamination of 5-methylcytosine to thymine. Consequently, S1 represents a clock-like feature for cancer tissues, termed mitotic clock, and may be correlated with the age of the individual^20^. SBS1 is usually co-observed with SBS5, as seen in our HMR cases. SBS5 is another clock-like signature that correlates with the individual’s age but not with the mitotic clock. The etiologic factors causing SBS5 are uncertain, but the effect of tobacco smoking is suspected^19^, interestingly, nine of the 10 cases in our current study were tobacco smokers. We observed 10% contribution of SBS26 to the mutational profile of HMR samples (Fig. 2a, light blue). Defective DNA mismatch repair and microsatellite instability (MSI) contribute to SBS26^21^.

The presence of SBS26 in the HMR group led us to investigate microsatellite instability (MSI) using MSI sensor on paired normal and tumor sequence data^22^. This analysis demonstrated high MSI scores (%15) in tumor samples with SBS26 signature (Table 2). The presence of SBS26 was highly correlated with the MSI scores (r^2^ = 0.9908) (Fig. 2c).

**Table 2:** Percent signature 26 and microsatellite instability in AA ESCC.

Tumor	% S26	%MSI
T1	9	15.3
T5	11	15.9
T6	11	15.0
T7	10	15.3
T8	10	15.1
T14	10	15.6
T9	0	0.4
T15	0	0.2
T16	0	0.1
T19	0	0.0

In LMR samples, we found other mutational signatures such as SBS18, possibly due to DNA damage by reactive oxygen species in two tumor samples (T9 and T15), SBS4, due to tobacco smoke carcinogens in three tumor samples, and SBS8, possibly due to defective homologous recombination repair in a tumor sample from a female ESCC patient (Fig. 2b).

### SCNAs in AA ESCC genomic regions encode cancer-related genes

**Figure 3:**
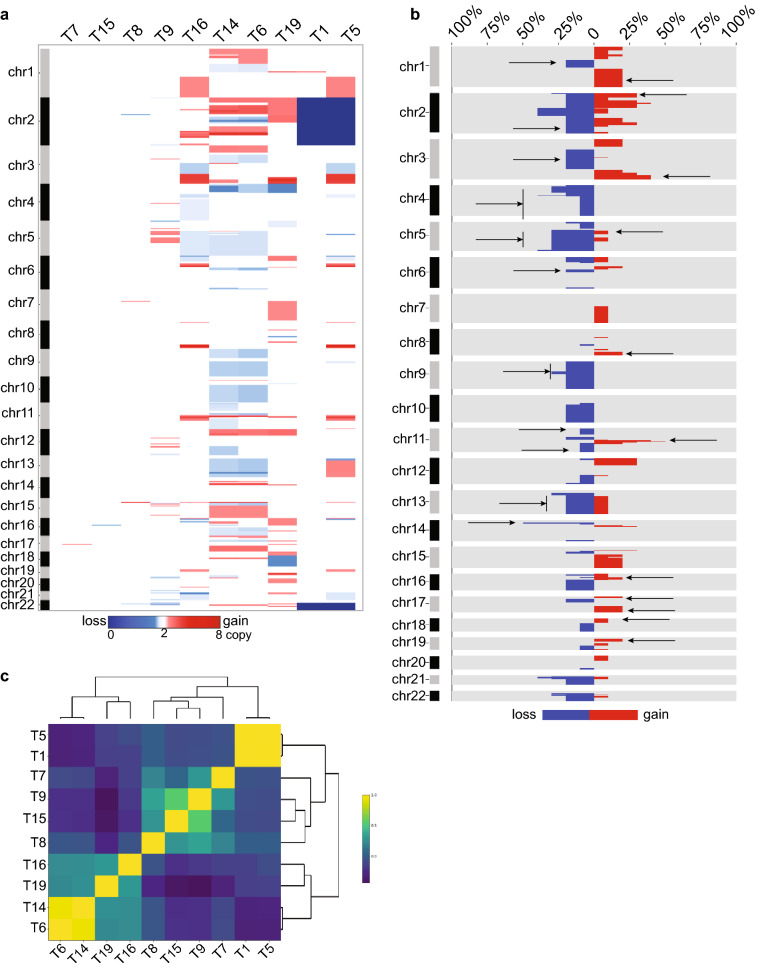
SCNAs in AA ESCC genomic regions encode cancer-related genes. (**a**) SCNA region profiles by 1 MB were visualized using CNApp. Dark blue represents homozygous deletion (copy number 0), white represents diploid (copy number 2), red represents copy number gains (the darkest red is copy number 8). (**b**) SCNA region frequency plot shows the percentage of samples with gains (red), losses (blue), and no alteration (gray) across chromosomes. Arrows represent the regions that also found in the 17 AA ESCC cohort analysis. (**c**) Plot shows the hierarchical clustering for samples correlation by Pearson correlation. Pearson *r* correlation coefficient *r* = 1 is yellow, *r* =  − 1 dark navy color.

Our prior CGH analysis on 17 AA ESCC tumors revealed a preponderance of gains and losses in multiple chromosomal regions suggesting the involvement of a plethora of SCNAs^17^. In the present study, we evaluated SCNAs in an orthogonal panel of tumor samples from mostly late-stage AA patients with ESCC. Multiple regions of copy number gain and loss were observed in seven samples: T1, T5, T6, T9, T14, T16, and T19 (Fig. 3a and Supplementary Fig. S2). These SCNA-rich samples displayed on an average of 62 (ranging from 46 to 90) different chromosomal regions. Tumor samples (T1, T7, T8, and T15) with fewer copy number aberrations showed on average 15 SCNAs (ranging from 5 to 25), presenting fewer copy number aberrations. Sample T5 and T9 had a moderate number of SCNAs (Fig. 3a).

The most recurrent copy number changes along the AA ESCC genome were seen in the amplification of chromosomes 3q, 8q, 11q, and 12p (Fig. 3b). Three tumor samples (T5, T16, and T19) exhibited high-level amplification in 3q26, including *WWTR1*,* TP63*,* PIK3CA*,* SOX2*,* SOX2-OT*,* and ZNF639* genes (Fig. 3b and Supplementary Fig. S3a, b). We also observed an extended 3q amplicon, a distinct ESCC1 feature, in 30% AA ESCC patients. The region 8q harboring the proto-oncogene *MYC* was amplified in T5, T14, and T16 (Fig. 3b and Supplementary Fig. S3b). One of the highly amplified regions in chromosome 11q13 encoding several critical genes, including *CCND1*,* FGF3*,* FGF4*, was observed in T5, T6, T14, T16, and T19 (Fig. 3b and Supplemental Fig. S3a, b). Samples T14, T19, and T6 revealed amplification on the short arm of chromosome 12, including 12p12.1 that harbors *KRAS*,* SOX5*,* ARNTL2*,* BCAT1* (Supplementary Fig. S3b).

T16 displayed the highest copy number amplification with eight copies of the 2q33.1 region encoding *CASP10* and *CFLAR*, both of which function in cell death^23^. The second-highest amplification number with seven copies of five different chromosome regions encode for genes including *SOX2*,* ANO1*,* FADD*,* CTTN*,* POLD3*,* RNF169*,* XRRA1*,* PAX9* in sample T19 in this study (Supplementary Table S3). A rare, distinctive homozygous deletion of the entire chromosome 2 was displayed by T1 and T5 samples (Supplementary Fig. S3a). T1 and T5 additionally displayed a deletion of 22q (Supplementary Fig. S3a). Chromosome 22q harbors several cancer genes, including histone acetyltransferase *EP300* (E1A Binding Protein 300), a known ESCC driver gene^14^. Two tumor samples, T14 and T19, displayed a loss of 4p13 that encodes *RHOH*, a member of the RAS superfamily, and *PHOX2B*, a homeobox transcription factor (Supplementary Fig. S3b). Our previous CGH analysis revealed the complete loss of chr4 in all 17 ESCC samples (Fig. 3b)^17^. Additionally, T19 carries a deletion of 18q21 harboring *SMAD4*, a region that we found deleted in another set of AA ESCC tumors^24^. Amplification on the short arm of chromosome 2 that contains a super-enhancer *ZFP36L2* gene was observed in three samples (T6, T14, and T19 ) and amplified in 30% of AA ESCC tumors in our previous aCGH study.

Chromosomal copy number gains in 1q distal, 2p proximal, 3q distal, 5p proximal, 8q distal, 11q, 16p, 17p and 17q, 18p, 19p were also found in 17 AA ESCC cohort^17^. Chromosomal copy number losses, also observed in 17 AA ESCC cohort, included 1p distal, 2qdistal, 3p distal and 3q proximal, whole chr4, whole 5q, 6q proximal, 9p distal, 11p proximal, 11qdistal, 13q, 14p proximal regions. The regions also found in 17 AA ESCC cohort indicated by arrows in Fig. 3b.

Although each patient’s tumor sample revealed a unique set of genomic alterations, a clear pattern of shared regions of SCNA loci between tumors was evident in the tumor samples from the ten patients. The correlation analysis by Pearson’s method demonstrated two hierarchical clusters of AA ESCC according to their SCNA profile (Fig. 3c). Copy number-rich samples T6, T14, T19, and T16 having more common SCNAs were clustered together and formed Cluster 1. Except for T16, Cluster 1 samples showed 12p amplification, a region that encodes *KRAS*. The second cluster, Cluster 2, contained samples with fewer copy number changes and is further divided into subclusters. Cluster 2A contained tumor samples T7, T8, T9, and T15, which harbored mostly focal copy number changes. Cluster 2B was formed by samples T1 and T5 containing broader changes such as deletion of chromosomes 2 and 22.

### Rare mutation and amplification in *EGFR* gene

**Figure 4:**
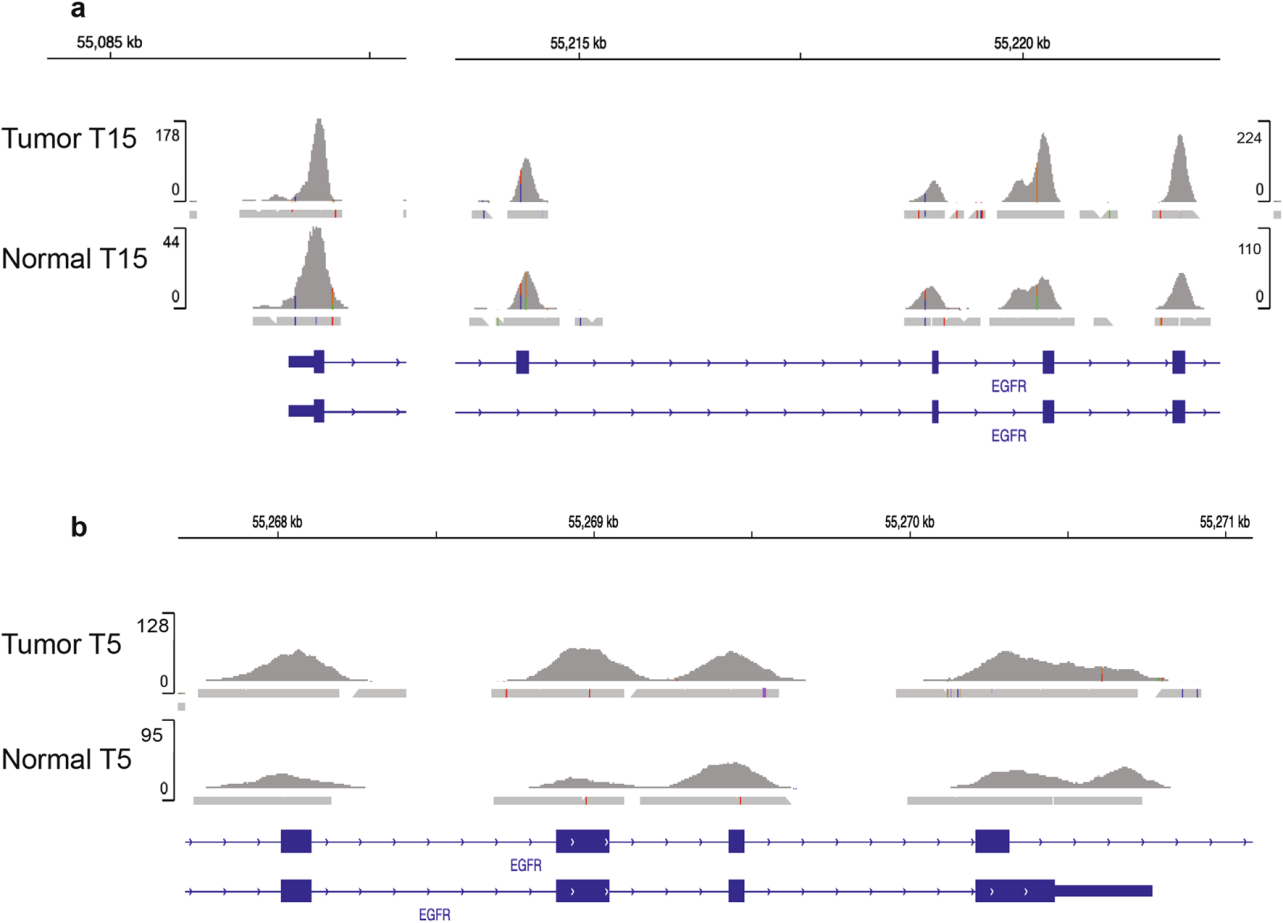
Amplified mutant EGFR in AA ESCC. (**a**) *EGFR* amplification in Sample T15 and corresponding normal sample sequencing readouts visualized in Integrative Genomics Viewer. (**b**) *EGFR* amplification in Sample T5 and corresponding normal sample sequencing readouts visualized in Integrative Genomics Viewer.

Two tumor samples, T15 and T5, carried *EGFR* mutations and amplification. Sample T15 harbored *EGFRvIII* mutation caused by a deletion of exons 2–7, in addition to an amplification of the region, chr7:55087058-chr7:55223523 on which *EGFR* gene is located (Fig. 4a). Sample T5 was found to carry *EGFR vIVa* which represents deletion of exons 25–27, and amplification in chr7:55268106-chr7:55272949, which includes *EGFR* (Fig. 4b). In samples T5 and T15, we observed a mutual exclusive amplification pattern between *EGFR* and *KRAS* genes. However, amplification of cell cycle-related genes and *MYC* co-occurred with *EGFR* and with *KRAS* amplification.

### Single nucleotide variations and short InDels

**Figure 5:**
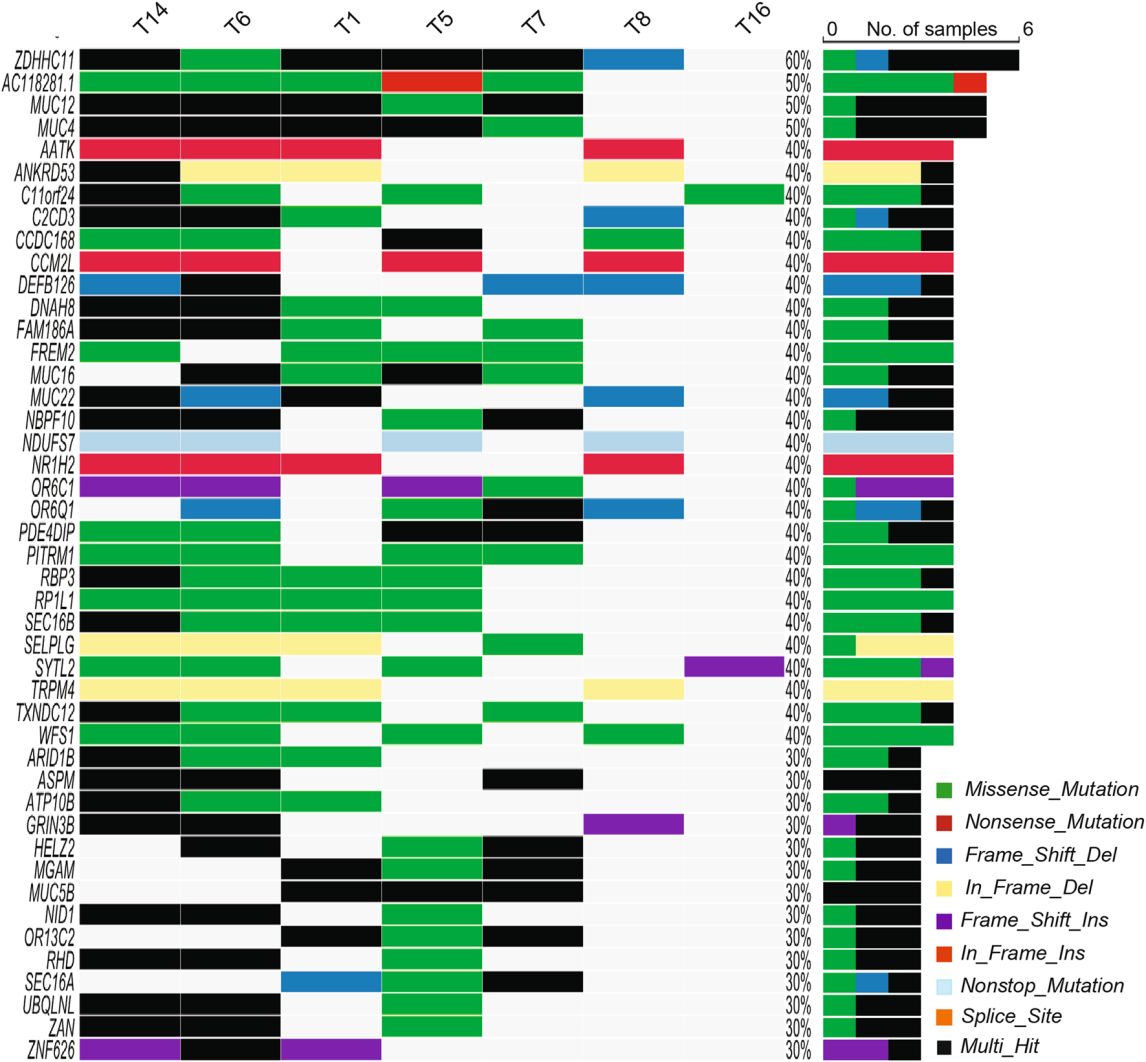
Single nucleotide variants of AA ESCC. Oncoplot by maftools was visualized mutations of missense, frameshift InDels, nonsense, splice site and translation start site that occurred in at least 30% of the tumors. Top 45 mutated genes were plotted.

The high mutation burden in half of our AA tumor samples led to the detection of mutations in 6169 unique genes of which approximately 50% carried one mutation per gene. Analysis of frequently mutated genes showed that the top 45 genes were found in seven tumor samples (Fig. 5). The most frequently mutated gene in six samples was *ZDHHC11* (zinc finger DHHC-type containing 11) located at 5p15.33. Although the gene carried various types of mutations within the same sample, we suspected the passenger nature of these mutations in *ZDHHC11* partly due to scarce reports on mutated *ZDHHC11* in cancer and the potentially benign consequence of these mutations. The frequency-based identification of significant genes was not possible because of the small size of our cohort and the high mutation rate in some of the tumor samples.

**Figure 6:**
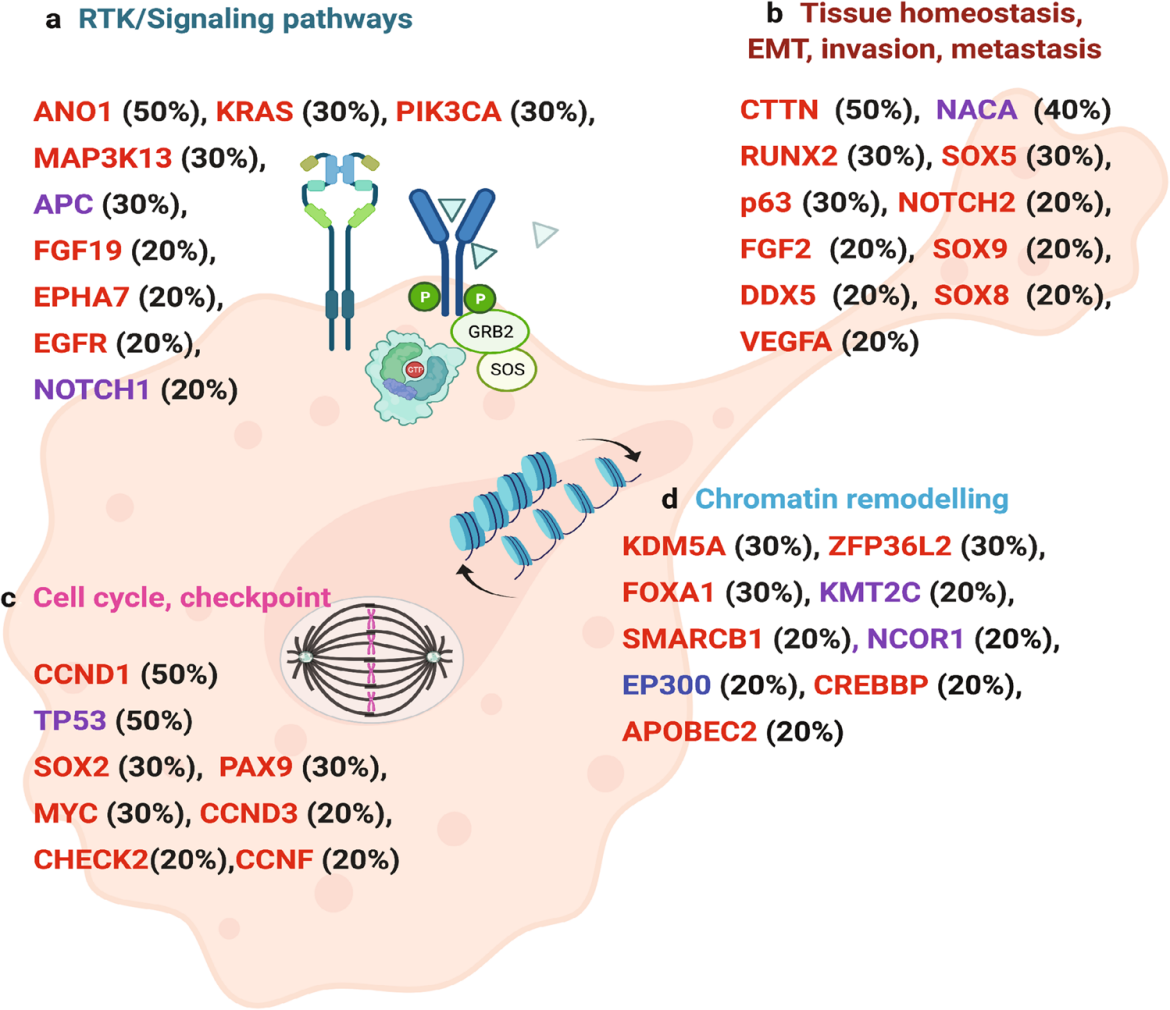
Summary of significantly altered genes in AA ESCC. (**a**) Genes that were mutated, or amplified in proliferation, cell cycle and cell cycle checkpoint controls. (**b**) Amplified genes found in squamous cell homeostasis, epithelial to mesenchymal transition (EMT), invasion and metastasis. (**c**) Genes that were amplified or mutated in receptor tyrosine kinase (RTK) and signaling pathways (**d**) Chromatin remodeling genes that were amplified, deleted, or mutated. Red designates amplification, blue indicates deletion, and purple shows missense mutations. Figure was created with BioRender.com.

To prioritize cancer-related genes in AA ESCC, we employed a combination of analyses in Opencravat^25^. We identified 23 missense and splice site mutations and 34 noncoding or synonymous variants previously described in ESCC in COSMIC database (Supplementary Table S4). The most notable ones were TP53 p.Ile232Asn and ZFP36L2 p.Ser105Leu, both of which may be damaging as predicted by Combined Annotation-Dependent Depletion (CADD)-Phred score of 31 and 24.6, respectively. Further analyses with multiple tools, such as CADD^26^, and annotation of deleterious genetic variants using neural networks (DANN)^27^ and maftools^28^ revealed damaging mutations in protein coding regions. These variants were overrepresented in several genes involved in various pathways including WNT, NOTCH signaling, TP53 tumor suppressor pathway, apoptosis, cell to cell communication and cell motility (Fig. 6 and Supplementary Tables S5 and S6).

We then searched for significant mutually exclusive or co-occurring pairs of genes (Supplementary Fig. 4). We did not detect any significantly mutually exclusive gene pairs. Among 23 co-occurred pairs which we detected in our current AA ESCC tumor samples, mutant *MUC4* and *MUC12* pairing is interesting as this pair was previously observed in smoking-associated non-small cell lung cancer patients^29^. This finding may illustrate the mutagenic role of tobacco in AA ESCC.

### Candidate cancer driver genes in AA ESCC

To predict candidate cancer driver genes and prioritize cancer-related genes in AA ESCC, we employed a combination of analysis algorithms that included CancerGenomeInterpreter (CGI), 20/20 +, CHASMplus, and dNdScv. These tools predicted *TP53* (p.Ile232Asn), *NCOR1* (p.Leu2168Phe), *APC* (p.Ser338Tyr), *KMT2C* (p.Gly315Ser), *CDKN1B* (p.Met52Arg), *NOTCH1* (p.Arg353Cys) as possible drivers (Supplementary Table S7).

In addition to the DNA binding domain mutation (p.I232N) of *TP53*, a mutation in Chr17.g.7576852:C > T led to the splice site loss creating an intron inclusion between exons 9 and 10 in two samples (T5 and T16). In T5, a second *TP53* splicing mutation (chr17.g.7673534:C > T) which is SNP rs11575997, was previously reported in various cancers in the COSMIC database, including five esophageal cancers. This variation creates a splice donor site mutation at exon 10 and is predicted to have a damaging effect on the protein, suggesting a pathogenic impact (https://www.ncbi.nlm.nih.gov/clinvar/RCV000785504.1). This variation creates a splice donor site mutation at exon 10 and is predicted to have a damaging effect on the protein, suggesting pathogenic impact (https://www.ncbi.nlm.nih.gov/clinvar/RCV000785504.1).

In summary, the current study identified two subgroups of ESCC based on tumor mutation burden (TMB), recurrent mutational signatures in HMR samples, and diverse signature profiles in LMR samples. We also detected MSI in HMR cancers. Our study revealed recurrent novel SCNA in cancer-related genes. SNVs and SCNAs suggested disruption of multiple pathways, including proliferation, cell cycle, epithelial to mesenchymal transition (EMT), invasion, and tumor metastasis in AA ESCC tumors (Fig. 6).

## Discussion

ESCC in African Americans has high mortality rate. Despite the high morbidity and mortality associated with this disease, there is a paucity of genomic studies in AA ESCC. Previously, we laid the groundwork for investigating AA ESCC which revealed a high rate of genomic imbalances^17^ and a dysregulated expression of genes in specific pathways^18^. The current study unmasked a multifaceted genomic landscape that defines a range of genomic alterations, and novel mutated loci that may potentially contribute to AA ESCC tumorigenesis.

We have conducted whole exome sequencing on 10 pairs of ESCC tumor and control tissues in AA, which to our knowledge, is the first comprehensive analysis of functional units in the ESCC genome exclusively in this ethnic group. Our results represent an important advance toward understanding the molecular basis for the lethality and aggressiveness of ESCC among African Americans.

AA ESCC displays a complex mutational profile that clustered half of our samples into a high mutation group, the other half formed a low mutation group. A similar distribution of high and low mutation rates has been reported in Sub-Saharan African ESCC patients^30^. Also, the former group exhibited a higher rate of mutation than the rates observed in Caucasian, Chinese, and Vietnamese ESCC patients^14,31^. Taken together, these findings are consistent with the high heterogeneity reported in ESCC tumors in patients of different ethnic origin.

An important novel finding in the current study is the identification of two EGFR mutations, *EGFRvIII* and *EGFRvIV*, accompanied by amplification in two AA ESCC tumor samples. While amplification of wild type *EGFR* has been previously reported in 7% of ESCC samples^32^, *EGFR* mutations have been rarely reported in ESCC^33^. Both *EGFRvIII* and *EGFR vIVa* mutations lead to constitutive EGFR signaling, tumor growth and progression pathways^34–36^. These *EGFR* mutations along with amplification are characteristics of Glioblastoma multiforme^37^ and wild type *EGFR* amplification was observed in ESCC with 6% frequency and associated with poor prognosis^38^. Consistent with previous reports, none of our AA ESCC tumor samples that displayed amplified mutant *EGFR* showed wild type *KRAS* amplification. The mutual exclusivity of *EGFR* amplification and *KRAS* mutations has been described in lung adenocarcinoma and colorectal carcinoma (CRC) and co-expression caused cytotoxic effect on cells^39,40^. Even though the incidence of *KRAS* amplification compared to its mutation is low, such as less than10% of patients with gastric cancer or CRC and 17% in EAC, clinical features of patients with *KRAS* amplification are distinct, and the amplification is usually associated with poor prognosis in these patients^41–44^. The oncogenic effect of wild type *KRAS* amplification is mediated through the increased receptor tyrosine kinase-dependent activation of the Ras pathway in CRC^45^. A recent study in Asian ESCC shows a 6% wild type *KRAS* amplification and its mutual exclusivity from *EGFR* amplification^38^. Like in gastric cancer, EAC, and CRC patients, *KRAS* amplification is associated with worse survival in ESCC patients^38^. Similar to mutant *KRAS*, wild type *KRAS* amplification confers EGFR inhibitor resistance^41^. Therefore, ESCC patients may benefit from detailed *EGFR* profiling and determination of tumor *KRAS* amplification status before targeted therapies.

Amplification in 3q26 harboring *PIK3CA* and SOX2 was observed in 30% of the current AA ESCC cohort and reported in 40% AA ESCC by aCGH^17^. *PIK3CA* amplification is associated with poor prognosis in curatively resected patients with ESCC^46^ and resistance to chemotherapy in other cancers^47^. *MYC* oncogene amplification was also observed in 30% of the AA ESCC tumor samples. MYC amplification is shown to be associated with lymph node metastasis and poor prognosis in ESCC^48^. *PIK3CA* and *MYC* amplifications co-occurred with either *EGFR* as observed in one sample or *KRAS* amplification in two samples in this study. The current study demonstrated 11q13 amplification, which is consistently amplified in 88% of AA and 68% of Asian patients^17,49,50^. Amplification of 11q13 is also revealed in a subset of HPV negative head and neck, lung, and esophageal squamous cell carcinoma^51^ and breast cancer^52^. Recurrent amplification of 11q13 is associated with nodal metastasis in ESCC and poor prognosis in head and neck cancer patients^49,53^. Critical genes encoded by this region include *CCND1*,* FGF3*,* FGF4*, which are involved in cell-cycle regulation and tumor cell proliferation in oral squamous cell carcinoma, hepatocellular carcinoma, and ESCC^11,54–56^.

Interestingly, 17p deletion, which is shared across all cancers, is rarely observed in African ancestry ESCC patients. Previously, we reported chromosome 17 gains in 17 AA with ESCC patients^17^. Another study on 51 South African ESCC cases did not display regional losses on chromosome 17^57^. However, other studies on Asian ESCC showed 17p deletions or losses up to 75% frequency^58,59^, suggesting that chromosome 17 imbalance profile could be a distinguishing feature for ESCC between ethnic groups.

In our dataset, homozygous deletion of the entire chromosome 2 and 22q in two AA ESCC patients was consistent with our previous CGH findings^17^. Chromosome 2 encodes 21 cancer hallmark genes^60^. The regions of deletion included several loci, including those coding for *LRP1B* (LDL receptor-related protein 1B) on 2q22.1-q22.2 and *NFE2L2* (nuclear factor, erythroid 2 like 2) on 2q31.2, both of which have been implicated in ESCC carcinogenesis^61,62^. Loss of chromosome 4p detected in the current study has been in AA ESCC samples^17^. The deletion of *SMAD4* in 18q21 is rarely found in ESCC in other ethnic datasets, albeit most frequently observed in EAC^63^.

Ethnicity-based differences in mutation frequency in specific genes are observed in ESCC^14^. For example, *TP53*,* EP300*, and *NFE2L2* showed a significantly higher mutation frequency in Asian ESCC patients (ESCC1) than Caucasians (ESCC2)^14^. The most commonly mutated gene in ESCC is *TP53* (more than 70% of all samples) in all other cohorts^11^. In the current study, *TP53* was found to be mutated in 50% of the patient samples. However, none of these mutations were located in recognized ESCC hotspots.

Fractions of mutations found in each trinucleotide context constitute a mutational signature profile that may infer mutational processes and etiologic agents leading to tumorigenesis^19^. Signature SBS16, the most frequent mutation signature in our cohort, is significantly correlated with alcohol consumption in liver cancers, head and neck squamous cell carcinoma, and ESCC, leading to a high mutation burden^64–69^. However, the dominance of Signature 16 among other signatures in our dataset is surprising; in more than half of the samples, Signature 16 is the predominant signature, comprised 60% of the total mutational profile. This might suggest the strong mutagenic effect of alcohol in AA ESCC patients. Interestingly, we did not observe the APOBEC signature, which is more frequently seen in ESCC tumor samples^70,71^. Further studies should focus on the molecular mechanisms of alcohol consumption by illuminating the direct effect of alcohol and alcohol metabolites on ESCC especially in AAs, since this may explain the increased frequency of ESCC in AA population. SBS16 may represent defects in nucleotide excision repair and increased DNA damage. Our mutational analysis also identified SBS26, a mutational signature associated with DNA repair and microsatellite instability.

A limitation of our current study is the small sample size. Validating these findings in a larger sample collection is necessary and warranted. Another limitation is that, with one exception, all other samples were derived from advanced-stage tumors. Due to the small sample size and apparent high mutation rate, there is a possibility of false-positive calls. Therefore, we set our variant call threshold to a read depth of 50x, which may lead to the under-representation of rare and highly heterozygous calls. Future studies in large sample size and single-cell studies may resolve heterogeneity issues. Epigenetics studies such as DNA methylation and chromatin accessibility might also shed light on the tumorigenesis and prognosis of ESCC in African Americans.

In summary, our present study, which to our knowledge, is the first comprehensive exome sequencing analysis exclusively directed at AA ESCC has revealed intriguing and novel somatic alterations and copy number variations. The associated mutational signatures have not been previously described in this particular cohort and are of importance, molecularly and clinically as they may, in part, form the basis for the aggressive nature of ESCC in African Americans. Unraveling key genetic changes that are pivotal to inception, progression, and metastasis could generate novel early diagnostic markers and actionable targets for efficacious intervention and drug discovery. Future studies are needed to illuminate and validate these findings that could eventually contribute to Precision Oncology in AA ESCC.

## Materials and methods

### Materials

Whole-exome sequencing of matched tumor- and normal-cell DNA from endoscopic biopsies or surgical specimens from ten AA ESCC patients were performed. The staging of the patients was done according to the methods described in the American Joint Committee on Cancer Staging Manual^72^. The District of Columbia Veterans Affairs Medical Center Institutional Review Board approved this study. Written informed consent and the approval for publication of results was obtained from each patient before the procedure. All experiments were performed in accordance with relevant guidelines and regulations.

### DNA extraction and whole-exome sequencing

Genomic DNA extraction from frozen tissues was done using the MasterPure DNA extraction kit (Epicentre Technologies Corp., Madison, WI). To proceed to exon capture, we enriched the samples using Agilent SureSelect XT Human All Exon V6 + UTR kit (Agilent Cat. No.5190-8884). Genomic DNA was first cleaved to a size range of approximately 200 to 500 bp, and then sequencing adapters were attached to these fragments. Paired-end sequencing at a read depth of 100X was performed on the exome libraries using the Illumina HiSeq 4000 sequencer.

### Analysis of sequences

Bioinformatic analysis of paired-end sequences/reads (FASTQs) started with trimming Illumina adapter sequences in them using Trimmomatic-v0.36 and then verifying the trimmed read lengths and quality using FastQC-v0.11.7^73,74^. All passed the quality check and were aligned to the human reference genome with decoy sequences (human_g1k_v37_decoy.fasta.gz) as provided in the resource package of GATK v.3.7 using Burrow-Wheelers Aligner (BWA-MEM)^75,76^. The output binary alignment map (BAM) files were then processed through Picard-v2.18’s MarkDuplicates tool^77^. Base recalibration was performed using the GATK-v3.8’s Base Quality Score Recalibration tool^75^. For somatic analysis, the base recalibrated tumor and normal BAMs were fed to Mutect2 (GATK-v3.8)^78^ to call somatic variants at chromosome positions covered in the target bed (Agilent SureSelect V6 + UTR). The indel realignment of each tumor and normal BAMs pair were performed using Sentieon Realigner tool-v201711.05^79^ on the Seven Bridges Platform before calling variants from VarScan2^80^, and Strelka2^81^ to reduce the possibility of false SNVs calls arising due to alignment around InDels. GATK’s best practices does not recommend performing InDel realignment of BAMs before feeding them to Mutect2 as the tool already performs a better haplotype-based local reassembly of reads as default. However, since Varscan2 lacks such reassembly step and Strelka2 has a rather tier-based local reassembly step that is subject to the evaluation of properties for variant locus, a prior GATK InDel realignment would ensure the improvement in read alignments before they are fed to these two variant callers. The resulting somatic variant call files (VCFs) were hard filtered with read-depth greater than or equal to 50X and variant allele frequency (VAF) greater than or equal to 5% to account for tumor heterogeneity. The read depth threshold of 50X was chosen as that was the minimum average read depth across target bed regions for each tumor sample. At such a considerable read depth threshold in exome sequencing, one can go lower in VAFs to detect somatic variants from tumor samples against matched normal^82^. Somatic variants called by at least two methods were combined and annotated with variant effects using the snpEFF-v4.3t tool^83^, and analyzed downstream using OpenCravat-v2.2.2^25,84^. Rare variants that have less than 1% minor allele frequency (MAF) in African population were retained and rest filtered out. The alignment of reads was manually reviewed using BAM files of each tumor and matched normal samples in Integrative Genomics Viewer (IGV) to reduce the risk of false positivity^85,86^. SNV was visualized using Maftools oncoplot function^28^ in RStudio version 1.4.1106 by using R 4.1.0. To compare mutation load against TCGA cohorts, tcgaCompare function was used.

### Somatic copy number alterations

SCNAs were determined using the CNVKit-v0.9.3 tool in the SevenBridges Genomics interphase^87^. Using CNVkit’s Reference module, we created a reference coverage file from the double realigned normal BAMs that were realigned initially among themselves and then, with their respective tumor BAMs. Such a double indel realignment of normal would correct indel alignments at the germline sites that are common between them thereby reducing SNV artifacts around those sites as well and providing a clean data for downstream analysis. With CNVKit Reference dependent workflow, in addition to a Reference coverage, a target bed file of baited regions and an anti-target bed file of regions not included in the target bed were generated. These prerequisite files and re-aligned tumor BAM files were used by the tool to iterate for CNAs in each tumor sample. Further, SCNAs for all tumor samples were aggregated to check for both overlapping and novel SCNAs. CNAPP was used to quantify and plot the frequency of SCNAs and to analyze SCNAs clinical relevance^88^. Focal SCNAs were plotted using Karyoplot in RStudio-v1.1.463^89^. The sequencing readouts of frequently mutated oncogenes and tumor suppressor genes were manually inspected in the BAM file using the Integrative Genomics Viewer- Broad Institute (IGV)^85,86^.

### Tumor mutational burden

Tumor mutational burden was calculated using the total number of somatic coding mutations per sample^90^.

### Microsatellite instability

Microsatellite Instability (MSI) of tumor samples was determined by using MSI sensor in SevenBridges. Cutoff value for MSI is > 3.5%^22^. This analysis is designated for normal-tumor pairs.

### Mutational signature profile

In SevenBridges Genomics Interface Mutational Signatures—deconstructSigs 1.8.0 DeconstrucSig^91^ was used to identify mutational signatures based on nonnegative matrix factorization by creating a mutational profile and comparing the profile with predefined COSMIC mutation signatures^19^. Default parameters were used in the analysis.

### Driver gene prediction

We employed 20/20 + (v1.0.1)^92^ and CHASMplus, Cancer Genome Interpreter^93^, and dNdScv^94^ for driver gene prediction. In 20/20 + analysis, the number of simulations was increased to 100,000 and the 2020plus_100k.Rdata trained classifier was used to improve the prediction performance.

### Pathway and protein interaction analysis

Reactome database^95^ was used to analyze possible protein interactions among proteins encoded by mutated genes. Overrepresentation results were considered where *p* < 0.05 and FDR < 0.1 thresholds were satisfied.

### Regulatory approval and consent for publication

The District of Columbia Veterans Affairs Medical Center Institutional Review Board (DC VAMC IRB #07077) approved this study. Written informed consent and the approval for publication of results was obtained from each patient prior to the procedure.

## Supplementary Information

Supplementary Figures.

Supplementary Table S1.

Supplementary Table S2.

Supplementary Table S3.

Supplementary Table S4.

Supplementary Table S5.

Supplementary Table S6.

Supplementary Table S7.

## Data Availability

All sequencing data generated or analyzed during this study have been submitted for the National Cancer for Biotechnology Information Sequence Read Archive (SRA) repository with the submission number SUB9998664.
