# Supplementary material for: Exome sequencing identifies novel somatic variants in African American esophageal squamous cell carcinoma
Source: Sci Rep. 2021 Jul 20;11:14814. doi: 10.1038/s41598-021-94064-0 (PMC8292420; doi:10.1038/s41598-021-94064-0)
Supplement: Supplementary file 1 — Supplementary Figures. [file 41598_2021_94064_MOESM1_ESM.pdf]

Exome Sequencing Identifies Novel Somatic Variants in African American Esophageal Squamous Cell Carcinoma

Hayriye Verda Erkizan, Shrey Sukhadia, Thanemozhi G. Natarajan, Gustavo Marino, Vicente Notario, Jack H. Lichy, Robert Wadleigh

Supplementary Figure S1

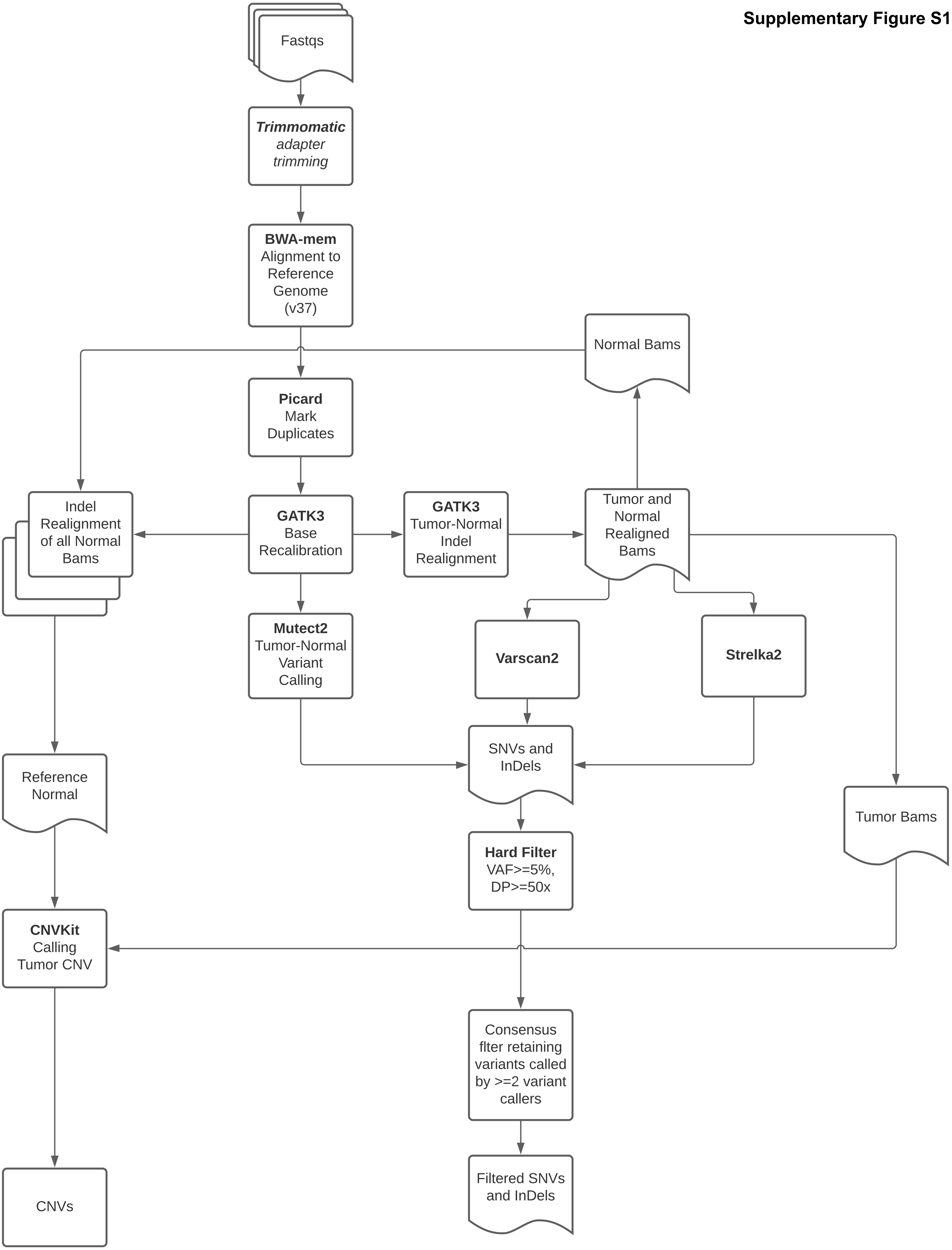

Supplementary Figure S1: Bioinformatic Analysis pipeline used in the study.
After preprocessing and mapping reads to the reference genome, somatic Single Nuclotide Variants (SNVs) and short InDels were called by using Mutect2, Varscan2 and Strelka 2. CNVKit was used for calling copy number variations.

Supplementary Figure S2

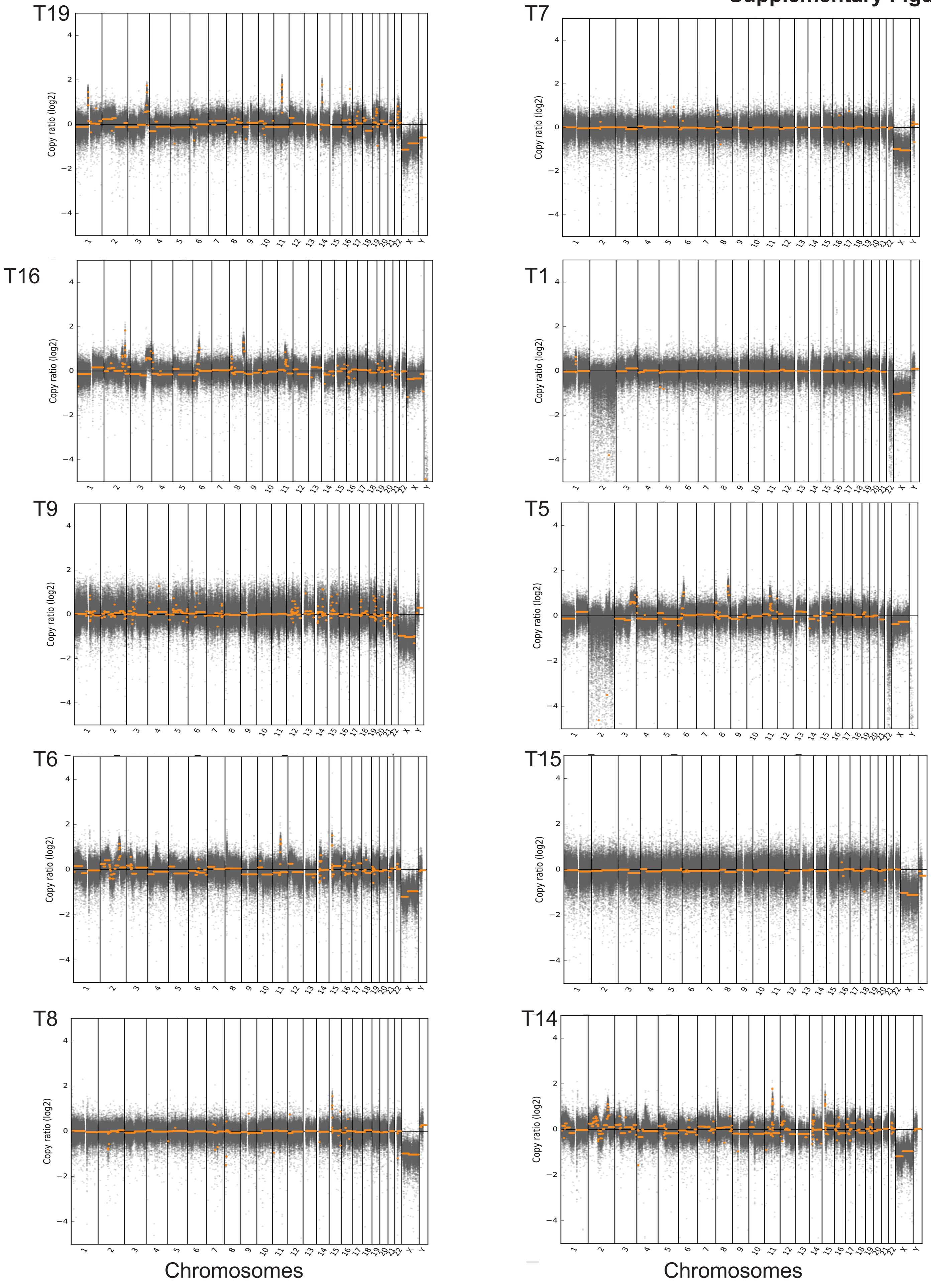

Supplementary Figure S2:Scatter plots of copy number aberrations in AA ESCC
The scatter plots of CNVKit results indicate the copy number changes across the genome. Y-axis represents copy number ratio, (log2).

Supplemenary Figure 3

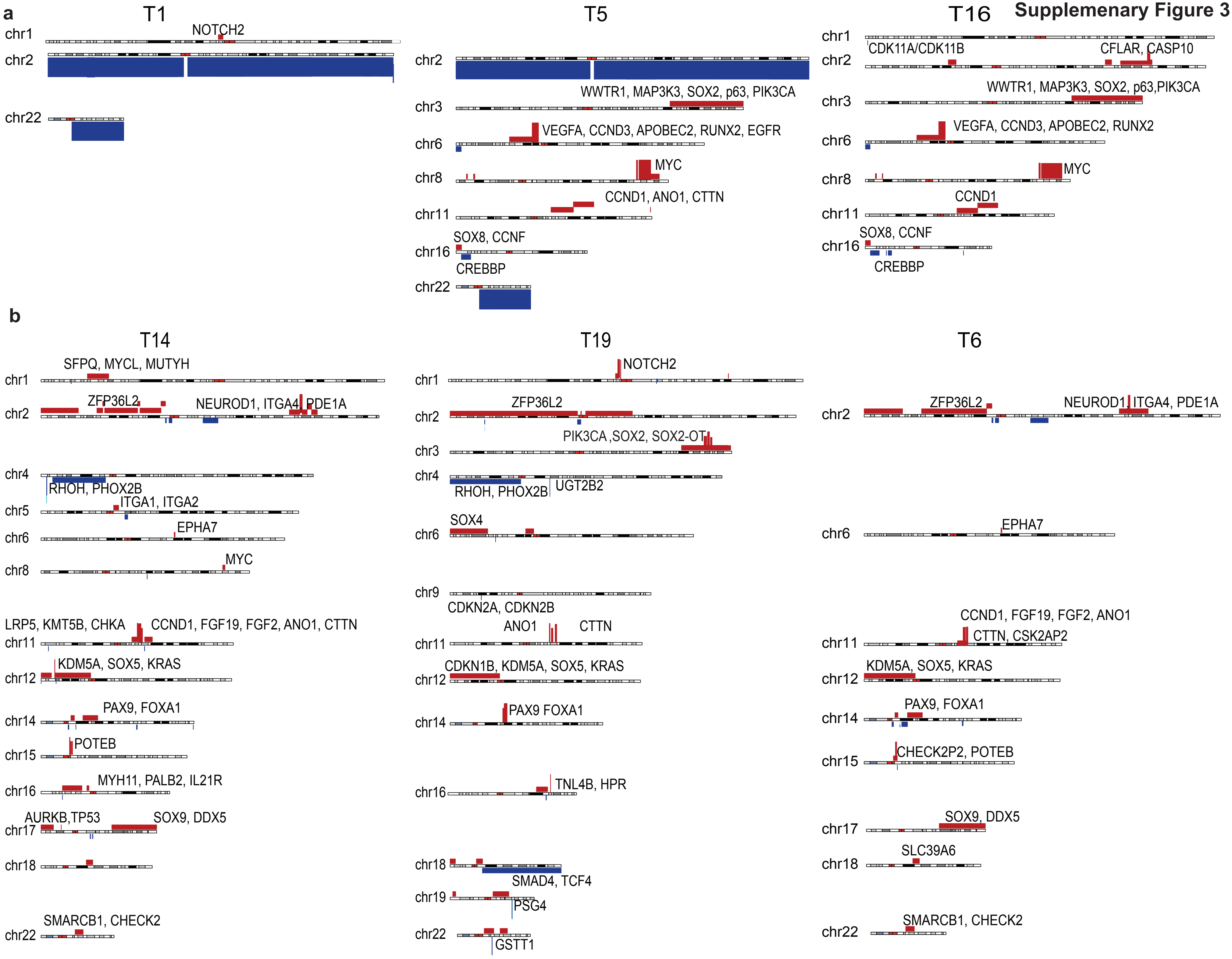

Supplementary Figure S3:Representative significant somatic copy number changes in AA ESCC
a. Samples T1 and T5 harbored whole chromosome 2 and chromosome 22q deletions.
b. SCNAs in AA ESCC genomic regions encode cancer-related genes.

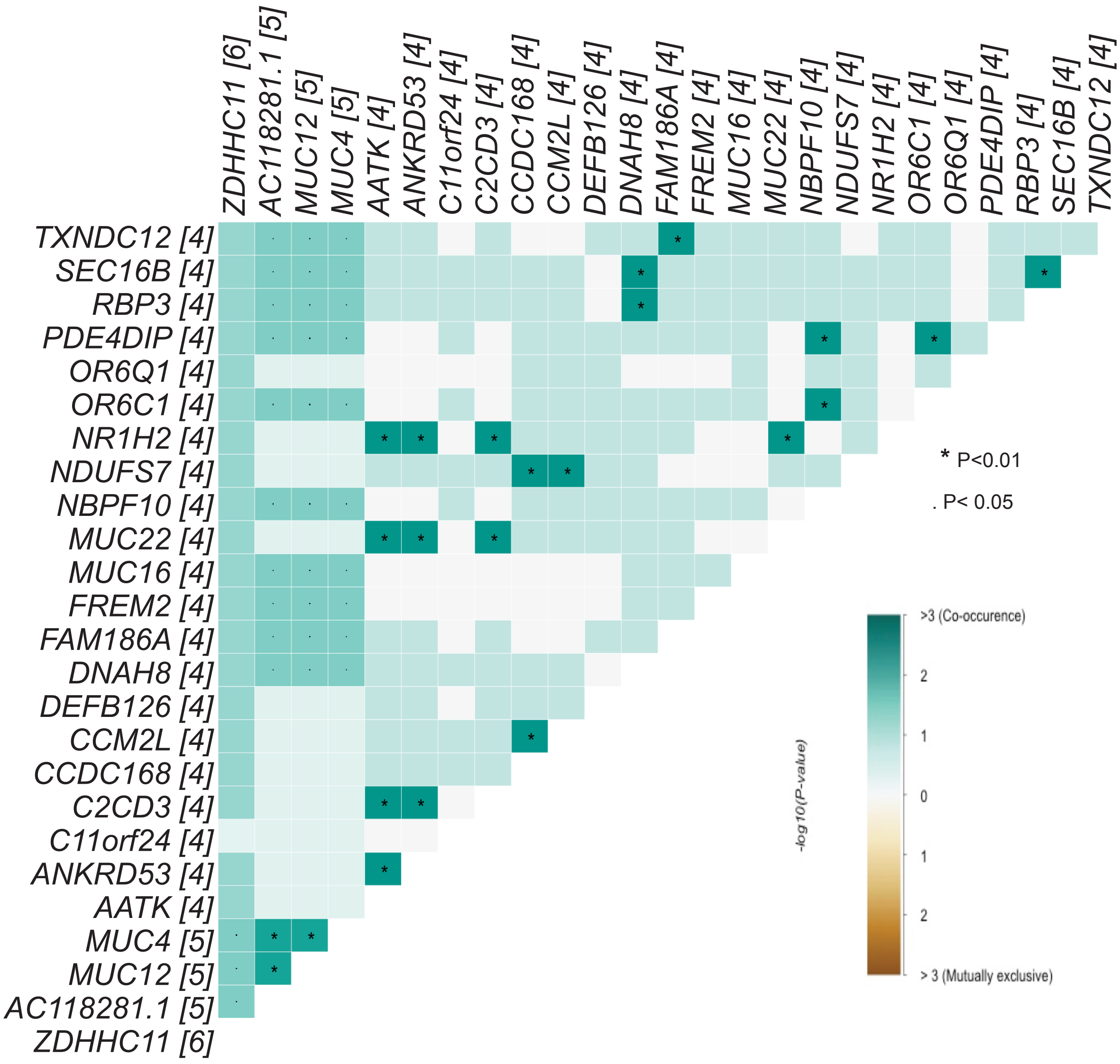

Supplementary Figrue S4: Co-occurance of mutations in AA-ESCC. The somatic interactions function of maftools detected co-occuring set of genes by performing pair-wise Fisher's Exact test.
